# Scientific overview of mandibular advancement devices: a bibliometric and altmetric analysis

**DOI:** 10.1590/2177-6709.31.3.e2625248.oar

**Published:** 2026-08-28

**Authors:** Karina CARDOSO, Isabela RAMOS, Aurélio de Oliveira ROCHA, Julia MULINARI, Julia Maldonado GARCIA, Carla Miranda SANTANA, Mariane CARDOSO

**Affiliations:** 1 Universidade Federal de Santa Catarina, Dental School (Florianópolis/SC, Brazil).

**Keywords:** Mandibular advancement, Orthodontic appliances, Activator appliances, Bibliometric, Altmetric, Avanço mandibular, Aparelhos ortodônticos, Aparelhos ativadores, Bibliometria, Altimetria

## Abstract

**Objective:**

To evaluate publications on mandibular advancement devices through bibliometric and altmetric analysis.

**Methods:**

A search was conducted in the Web of Science Core Collection (WoS-CC) initially in August 2024, with an update in December 2025, including all articles on mandibular advancement devices, with no restrictions on language or publication date. Editorials, abstracts, and conference papers were excluded. Two independent reviewers selected the studies and extracted data on citations, year, journal, study design, type of device, study objective, geographic origin, authors, and keywords. Altmetric analysis was performed using Dimensions. Spearman’s correlation tested associations between impact factor, year, and citations, and bibliometric networks were created with VOSviewer.

**Results:**

A total of 446 articles were published between 1979-2025. The most cited article received 352 citations, and the American Journal of Orthodontics and Dentofacial Orthopedics published the largest number of studies (n = 174). Clinical trials were the most frequent design (n = 175), mainly addressing skeletal, dental, facial, and esthetic changes (n = 175). The Herbst appliance was the most investigated (n = 129). Germany (n = 52) and Europe (n = 236) led the publications, with Giessen University as the most productive institution (n = 37). The most prolific author was Pancherz H (n = 31). Altmetric analysis revealed relevant mentions on Mendeley and Facebook.

**Conclusion:**

Europe concentrated the largest scientific output on mandibular advancement devices, particularly the Herbst appliance, in studies focused on skeletal, dental, facial, and esthetic changes.

## RESUMO

**Introdução:**

Avaliar publicações sobre dispositivos de avanço mandibular por meio de análise bibliométrica e altimétrica.

**Métodos:**

Uma busca foi realizada na *Web of Science Core Collection* (WoS-CC) inicialmente em agosto de 2024, e atualizada em dezembro de 2025, incluindo todos os artigos sobre dispositivos de avanço mandibular, sem restrições de idioma ou data de publicação. Editoriais, resumos e artigos de conferências foram excluídos. Dois revisores independentes selecionaram os estudos e extraíram dados sobre citações, ano, periódico, delineamento do estudo, tipo de dispositivo, objetivo do estudo, origem geográfica, autores e palavras-chave. A análise altimétrica foi realizada utilizando o Dimensions. A correlação de Spearman testou associações entre fator de impacto, ano e citações (SPSS), e redes bibliométricas foram criadas com o VOSviewer.

**Resultados:**

Um total de 446 artigos foram publicados entre 1979-2025. O artigo mais citado recebeu 352 citações, e o *American Journal of Orthodontics and Dentofacial Orthopedics* publicou o maior número de estudos (n = 174). Ensaios clínicos foram o delineamento mais frequente (n = 175), abordando principalmente alterações esqueléticas, dentárias, faciais e estéticas (n = 175). O aparelho Herbst foi o mais investigado (n = 129). Alemanha (n = 52) e Europa (n = 236) lideraram as publicações, com a Universidade de Giessen como a instituição mais produtiva (n = 37). O autor mais prolífico foi Pancherz H (n = 31). A análise altimétrica revelou menções relevantes no Mendeley e no Facebook.

**Conclusão:**

A Europa concentrou a maior produção científica sobre dispositivos de avanço mandibular, particularmente o aparelho Herbst, em estudos focados em alterações esqueléticas, dentárias, faciais e estéticas.

## INTRODUCTION

Class II malocclusion is the most prevalent malocclusion in orthodontic practice, affecting approximately 20-25% of the global population.^1^ Clinically, it is frequently associated with mandibular retrusion, increased overjet, compromised facial esthetics, and functional impairments, which may negatively affect patients’ quality of life.^2^

Although treatment with fixed appliances combined with intermaxillary elastics remains widely used, this approach is highly dependent on patient compliance and may limit treatment predictability and efficiency.^3^ In this context, mandibular advancement devices have gained substantial clinical relevance, as they promote continuous mandibular advancement with reduced reliance on patient cooperation, correcting skeletal and occlusal discrepancies.^3^ ^,^ ^4^ Also called Class II correctors, these appliances can be fixed or removable.^5^ Fixed functional appliances are further classified as rigid, flexible, or hybrid systems.^5^ Clinical studies and systematic reviews have demonstrated that these appliances can produce significant dentoskeletal changes, including overjet reduction, improvement in maxillomandibular relationships, and favorable aesthetic results, especially when used during periods of active growth.^4^

Scientific evidence suggests that mandibular advancement devices may exert effects beyond sagittal correction, influencing functional parameters such as airway dimensions and mandibular posture, reinforcing their importance in comprehensive orthodontic and orthopedic treatment.^6^ The growing clinical adoption of these devices is reflected by a reported 15% increase in their use for the treatment of Class II malocclusion.^7^

Orthopedic functional appliances that stimulate mandibular growth are primarily indicated for growing patients,^8^ including appliances such as the Bionator^9^ and Frankel II.^10^ In patients treated during or after the pubertal growth spurt, fixed functional appliances are more commonly indicated, including the Herbst,^11^ Twin Block,^12^ and PowerScope.^8^ More recently, removable systems such as the Invisalign Mandibular Advancement appliance have also been introduced for this age group.^13^ Although the magnitude of mandibular growth induced by mandibular advancement remains debated, a systematic review demonstrated that the Herbst and Twin Block appliances present greater growth efficiency per month, compared with other approaches.^4^

Bibliometric reviews are a method of quantitatively analyzing scientific publications in each area of knowledge, mapping and identifying gaps, assisting in the formulation of future research and evaluating the influence of published research, by counting publications and citations.^14^ Altmetric analyses are an alternative method of evaluating citations, where data are extracted and evaluated from online environment tools, making it possible to measure the social impact of academic research.^15^ Although bibliometric analyses have been conducted on various orthodontic topics^16^ and on alternative therapeutic approaches for the correction of Class II malocclusion,^17^ no study has systematically evaluated the scientific landscape related to mandibular advancement devices. Given their central role in the orthopedic and orthodontic management of skeletal Class II patients, as well as the substantial evolution in appliance design, biomechanics, and clinical indications over time, an in-depth assessment of the global research output in this field is needed. Bibliometric and altmetric approaches provide a robust framework to identify influential publications, research hotspots, collaborative patterns, and emerging innovations. Therefore, the aim of this study was to conduct a comprehensive global bibliometric and altmetric analysis of the literature on mandibular advancement devices.

## MATERIALS AND METHODS

This review was conducted in accordance with the Guideline for Reporting Bibliometric Reviews of Biomedical Literature (BIBLIO).^18^

An electronic search was conducted on August 18, 2024, and updated on December 20, 2025, in the Web of Science Core Collection (WoS-CC) database (*https://www.webofscience.com*). The search strategy was based on terms, keywords and synonyms from Medical Subject Headings (MeSH) related to the topic “mandibular advancement devices”, available in Supplementary File 1.

The study selection process was conducted in two stages. Initially, all retrieved records were screened by two independent reviewers (KC and IR) based on titles and abstracts. Articles that clearly did not address mandibular propulsion devices or the treatment of Class II malocclusion were excluded at this stage. In the second stage, the full texts of potentially eligible studies were independently assessed by the same reviewers, to confirm eligibility. Disagreements at any stage were resolved through discussion, and when consensus could not be reached, a third reviewer (AOR) was consulted.

Regarding eligibility criteria, all studies addressing mandibular advancement devices used for the treatment of Class II malocclusion were eligible for inclusion, with no restrictions on publication date, language, or study design. Original research articles, clinical trials, case/series report, observational studies, laboratory studies, systematic reviews, and narrative reviews were considered. Editorials, conference abstracts and letters to the editor were excluded. Duplicate records and irrelevant studies were removed prior to full-text assessment.

The following data from the included articles were collected and tabulated in Microsoft Excel^®^ 2010 (Microsoft, Redmond, WA, USA): number of citations in WoS-CC, year of publication, journal, impact factor (IF) (2024) (Journal Citation Reports from Clarivate Analytics), study design, themes (type of mandibular advancement devices used and main objective of the study), country and continent, institution (based on the corresponding author’s affiliation), author and keywords. The studies were classified according to the study design as: laboratory studies, observational studies, clinical trials, case reports/series, literature reviews and systematic reviews.

The Visualization of Similarities Viewer software (VOSviewer, version 1.6.17.0, Netherlands) was used to generate bibliometric networks to demonstrate the results found between authors, keywords, co-citation analysis and bibliographic coupling analysis. To interpret the network analysis, larger circles and letters with larger font represent results with greater occurrence. While smaller circles and smaller font demonstrate less frequent results. The lines that interconnect the terms demonstrate the collaboration between them.

The altmetric analysis was conducted in the free Dimensions application (*https://app.dimensions.ai/discover/publication*), in which the performance of articles on mandibular thrusters in the social environment was evaluated, through social networks, traditional media and online reference managers. The same search applied in the WoS-CC was applied in the Dimensions, and the selection of articles was carried out by two independent researchers (KC and IR). The following data were collected: Altmetric Attention Score (AAS), electronic address (DOI) and place where the article received the mention. The articles were organized in descending order, in relation to the AAS index.

The SPSS for Windows software (SPSS, version 26.0; IBM Corp) was used for data analysis. The normality of the data was tested using the Kolmogorov-Smirnov test. Due to the non-normal distribution of the data (p < 0,01), Spearman’s correlation test was used for the year of publication, journal impact factor (IF) and number of citations. The interpretation of the correlation test was conducted according to Mukaka.^19^

## RESULTS

### SEARCH RESULTS

**Figure 1:**
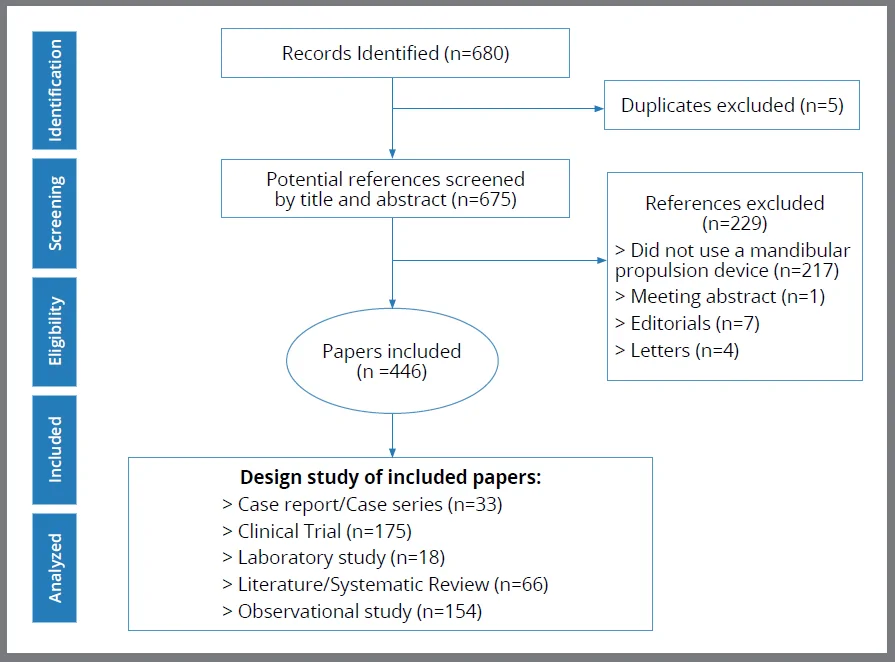
Flowchart of the study selection protocol.

The initial search resulted in 680 articles. After applying the eligibility criteria, 446 articles were included in this bibliometric analysis. Figure 1 illustrates the selection of articles. 

### YEAR OF PUBLICATION

**Figure 2:**
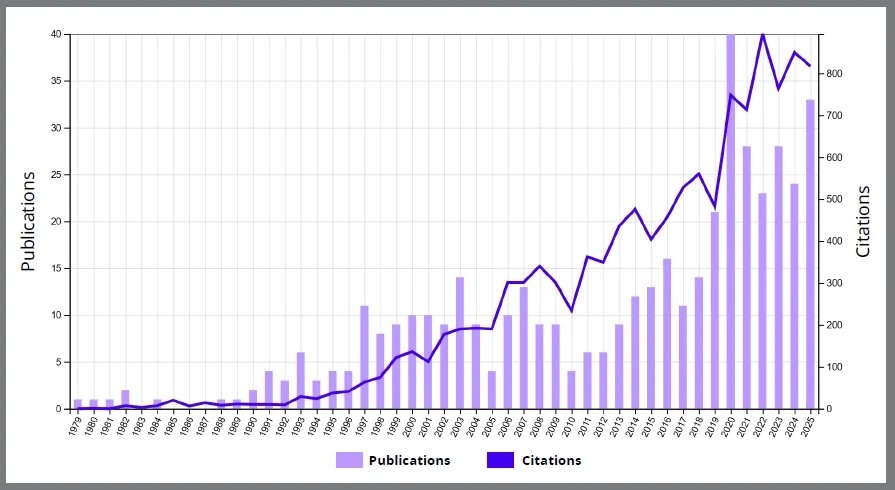
Distribution of the number of publications over the years.

The included articles were published between 1979 and 2025. The largest number of publications occurred from the 2000s onwards. Figure 2 illustrates the relationship between the year of publication of the articles and the number of citations. The oldest article is authored by Pancherz, H, published in 1979, with the title *“Treatment of Class II malocclusions by jumping the bite with the Herbst appliance. A cephalometric investigation”*.^11^ The most recent article is authored by Cukaj H.A. et al., published in 2025, entitled *“Mandibular advancement in dentofacial orthopaedics: effects on pharyngeal airway, dentoskeletal characteristics and quality of life: a randomised controlled trial”*.^20^

### CITATION ANALYSIS

In total, the selected articles received 11,809 citations in WoS-CC, of which 3,168 were self-citations, corresponding to 26.82%. The highest number of citations was 352, from the article *“The mechanism of Class II correction in Herbst appliance treatment. A cephalometric investigation”*,^21^ published in 1982. 

According to Spearman’s correlation, a weak positive correlation was observed between the number of citations and the journal’s impact factor (2024) (rho = 0.225) (p < 0,01) and strong negative correlation between the number of citations and the year of publication (rho = - 0.603) (p < 0,01).

### CO-CITATION ANALYSIS AND BIBLIOGRAPHIC COUPLING

**Figure 3:**
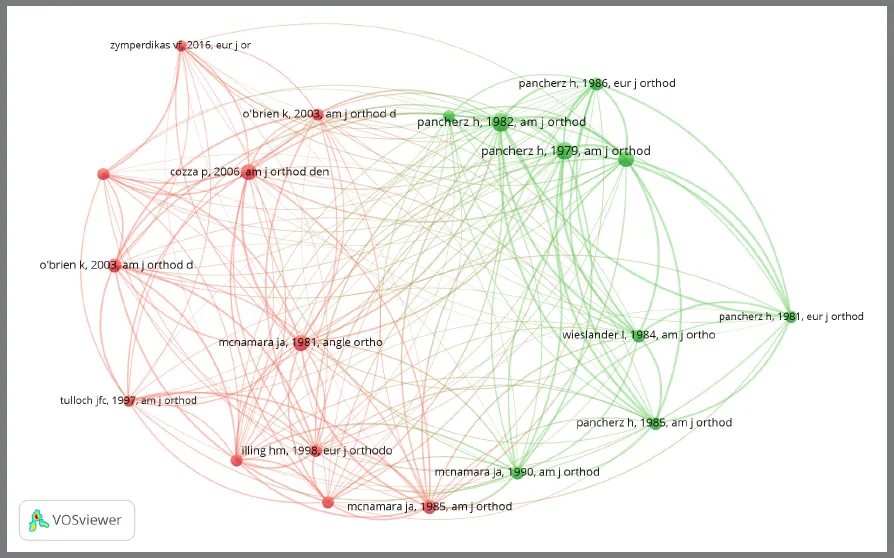
Co-citation network of cited references. For the analysis, cited references were considered the unit of analysis and the complete counting method was used. A minimum threshold of 41 citations was applied to include influential studies. Node size represents citation frequency, links indicate co-citation relationships, and colors denote groupings corresponding to the main conceptual and theoretical domains within the research field.

The co-citation analysis of cited references, performed using VOSviewer and illustrated in Figure 3, revealed two major intellectual traditions structuring the field. One cluster predominantly comprised studies addressing craniofacial growth, optimal timing of treatment, and the biological principles underlying functional orthopedic therapy, largely influenced by the seminal contributions of James A. McNamara and Tiziano Baccetti. The second cluster was mainly composed of clinical investigations on fixed functional appliances, particularly the Herbst appliance, with several influential studies by Hans Pancherz forming the core of this research line. Together, these clusters highlight the dual scientific foundation of the field, integrating biological concepts of craniofacial development with clinical approaches to mandibular advancement using orthopedic appliances. The bibliographic coupling analysis indicated a strongly interconnected research network, reflecting the extensive sharing of references among studies investigating mandibular advancement devices. Classic publications, including those by Hans Pancherz, Tiziano Baccetti, Paola Cozza, Kevin O’Brien, and John F. Tullock, showed stronger coupling relationships, highlighting their central influence in shaping the research landscape. In contrast, more recent publications tended to present weaker coupling connections, suggesting that emerging research topics are still developing and have not yet become fully integrated into the core knowledge structure of the field.

### JOURNALS AND IMPACT FACTOR

A total of 48 journals published articles on mandibular advancement devices. The journals, impact factor (2024) and number of publications are presented in Supplementary File 2. American Journal of Orthodontics and Dentofacial Orthopedics led the number of publications (n = 174; 39.01%), followed by European Journal of Orthodontics (n = 93; 20.85%) and Orthodontics and Craniofacial Research (n = 19; 4.26%). The journals with the highest impact factor (IF), based on Journal Citation Reports (JCR), were Cochrane Databases and Systematic Review (IF = 9.4), Journal of Dental Research (IF = 5.9), Progress in Orthodontics (IF = 5.0) and Journal of Clinical Medicine (IF = 4.9).

### STUDY DESIGN AND THEMES

The most used study design for this topic was clinical trials (n = 175; 39.24%), followed by observational studies (n = 154; 34.53%) and systematic reviews (n = 66; 14.80%).

Regarding the topics, when the studies were classified according to the type of mandibular advancement devices used (Theme I), the Herbst device was most frequently used (n = 129; 28.92%), Twin-Block (n = 92; 20.63%), Frankel FR-2 (n = 28; 6.28%), Forsus Device (n = 24; 5.38%), Bionator (n = 13; 2.91%), mandibular advancement aligner (n = 9; 2.02%), Jasper Jumper (n = 8; 1.79%), Mandibular Anterior Repositioning Appliance (MARA) (n = 4; 0.90%), Bass activator (n = 3; 0.67%), Carriere Motion 3D (n = 3; 0.67%) and multiple or unspecified propulsors (n = 133; 29.82%). 

Regarding the main objective of the studies (Theme II), most of the studies aimed to investigate dental, skeletal, facial and aesthetic changes (n = 175; 39.28%), followed by comparison between therapeutic approaches (n = 92; 20.63%), effectiveness of treatment with propulsors (n = 38; 8.52%), TMJ/condyle mandibular propulsive relationship (n = 31; 6.95%), assessment of the upper airways (n = 25; 5.60%), technical description (n = 24; 5.38%), assessment of mandibular growth (n = 20; 4.48%), stability of treatment (n = 17; 3.81%), report of patients’ experience (n = 9; 2.02%), alteration of masticatory function (n = 6; 1.34%), relationship between propulsion and sleep apnea (n = 5; 1.12%), association between mandibular propulsion and growth hormones (n = 2; 0.45%), quality of life during treatment with mandibular advancement devices (n=1; 0.22%) and evaluation of the cytotoxicity of mandibular advancement devices (n=1; 0.22%).

### COUNTRIES AND CONTINENTS

**Figure 4:**
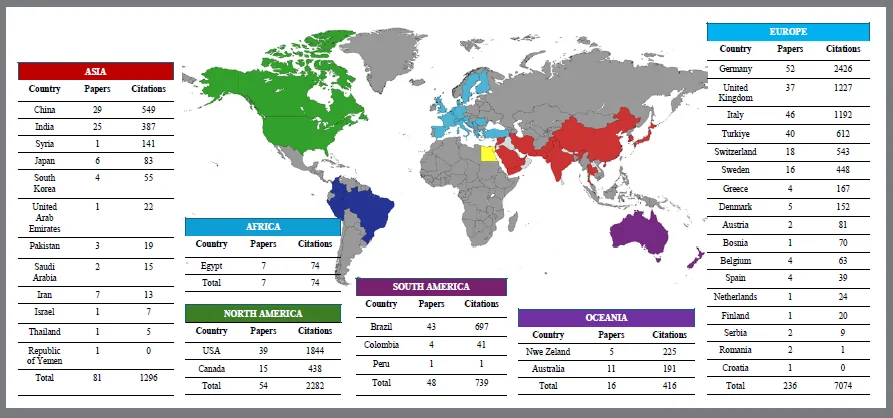
Worldwide distribution of the origin of publications on mandibular advancement devices.

In total, 37 countries participated in the publication of the 446 articles included in this bibliometric analysis, with the most prominent country being Germany (n = 52; 11.65 %), followed by Italy (n = 46; 10.31%), Brazil (n = 43; 9.64 %), Turkey (n = 40; 8.97%), United States of America (n = 39; 8.74%), and the United Kingdom (n = 37; 8.29%). The five continents participated in the publications on the subject, with Europe leading the publications (n = 236; 52.91%), followed by Asia (n = 81; 18.16%) and North America (n = 54; 12.10%). Figure 4 illustrates the continents and countries, with their respective numbers of publications and citations. 

### INSTITUTIONS

A total of 180 institutions had publications included in this research, with emphasis on the University of Giessen (n = 37; 8.29%) and University of São Paulo (n = 17; 3.81%). Table 1 shows the 10 institutions with the highest number of publications. In 18 articles, the authors were not affiliated with the educational institution and worked in private practice.

**Table 1:** Ten institutions associated with research on mandibular advancement devices.

Institution	Country	Number of articles
University of Giessen	Germany	37
University of Sao Paulo	Brazil	17
University of Manchester	United Kingdom	10
University of Alberta	Canada	10
University of London	United Kingdom	10
University of Michigan	United States	09
University of Hong Kong	China	08
University of Bern	Switzerland	05
Erciyes University	Türkiye	05
University of Otago	New Zealand	05

### CONTRIBUTING AUTHORS

**Figure 5:**
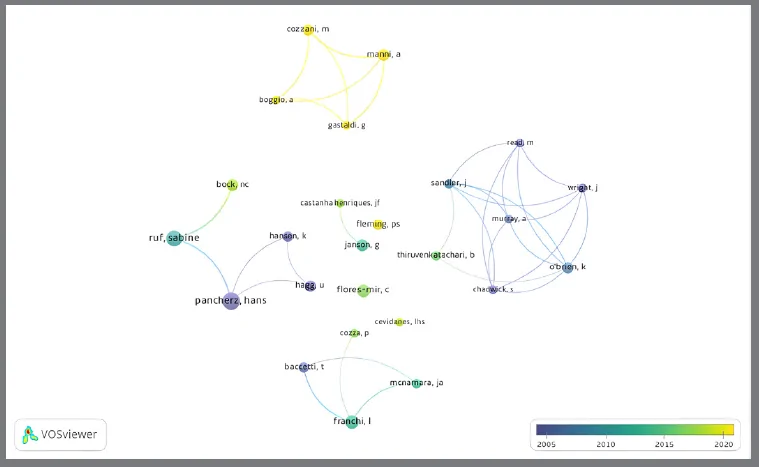
Main groups/authors who researched mandibular advancement devices. Minimum number of occurrences of the author: 6 studies. The names written in highlighted font and corresponding to the largest circles are associated with the most frequent authors. On the other hand, the names associated with smaller fonts and smaller circles correspond to the least frequent authors.

A total of 1,285 authors contributed to the 446 publications, with the author who published the most was Pancherz, H. (n = 31; 1,192 citations), followed by Ruf, S. (n = 25; 873 citations) and Franchi, L. (n = 17; 877 citations). Table 2 presents the 10 authors with the highest number of publications. The frequency with which the authors appear, and the co-authorship relationship are shown in Figure 5.

**Table 2:** Authors with the highest number of publications on mandibular advancement devices.

Authors	Number of articles	Number of citations
Pancherz, H.	31	1992
Ruf, S	25	873
Franchi, L.	17	877
Flores-Mir, C.	14	273
Manni, A.	13	86
Bock, N.C.	13	146
Janson, G.	12	177
O’Brien, K.	10	764
Cozzani, M.	10	57
Hagg, U.	10	251

### KEYWORDS

**Figure 6:**
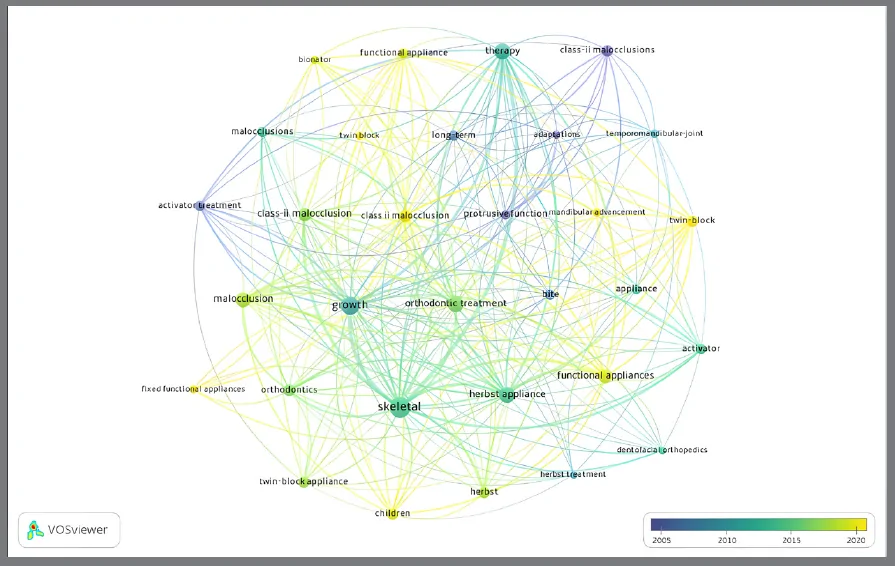
Frequency and interaction of the main keywords associated with the study. Minimum number of occurrences of the keywords: 17 studies. The larger dots are the keywords that appeared most frequently, on the other hand, the words that appear less frequently are associated with the smaller dots. The lines that join these dots indicate the relationship and the use of these words in the same studies. The keywords surrounded by blue dots indicate terms used by older manuscripts, and the most recent terms are represented by the color yellow.

The total number of keywords used by the 446 articles was 901, with the most prevalent being “skeletal” with 125 occurrences, followed by “growth” with 94 occurrences, and “Herbst appliance” with 78 occurrences. Figure 6 shows the most frequent keywords and the interconnection between them. 

### ALTMETRIC ANALYSIS

According to the Dimensions application, only 11 articles on mandibular advancement devices presented altmetric data, being referenced by social networks (X [former Twitter], Facebook and Blogs), traditional media (Wikipedia Page) and online reference managers (Mendeley). The complete list of articles is shown in Table 3. The article with the highest AAS score is entitled *“Occlusal stability of adult Class II division 1 treatment with the Herbst appliance,”* authored by Bock NC et al.^22^, published in 2010, in the American Journal Orthodontics and Dentofacial Orthopedics. This article received an AAS score of 5, with mentions in Blog (1) and Mendeley (79).

**Table 3:** Articles that featured altmetric performance.

Altmetric Attention Score (AAS)	Article	Mentioned by
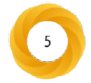	Bock NC, von Bremen J, Ruf S. Occlusal stability of adult Class II Division 1 treatment with the Herbst appliance. Am J Orthod Dentofacial Orthop. 2010 Aug;138(2):146-51. doi: 10.1016/j.ajodo.2008.09.031.	Blog (1) Mendeley (79)
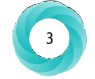	Ruf S, Pancherz H. Temporomandibular joint remodeling in adolescents and young adults during Herbst treatment: A prospective longitudinal magnetic resonance imaging and cephalometric radiographic investigation. Am J Orthod Dentofacial Orthop. 1999 Jun;115(6):607-18. doi: 10.1016/s0889-5406(99)70285-4.	Clinical Guideline Source (1) Mendeley (108)
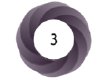	Bock N, Ruehl J, Ruf S. Orthodontic Class II:1 treatment-efficiency and outcome quality of Herbst-multibracket appliance therapy. Clin Oral Investig. 2018 Jun;22(5):2005-2011. doi: 10.1007/s00784-017-2294-9.	Wikipedia Page (1) Mendeley (39)
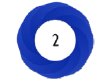	Lohrmann B, Schwestka-Polly R, Nägerl H, Ihlow D, Kubein-Meesenburg D. The influence of functional orthodontics and mandibular sagittal split advancement osteotomy on dental and skeletal variables--a comparative cephalometric study. Eur J Orthod. 2006 Dec;28(6):553-60. doi: 10.1093/ejo/cjl022.	Facebook Page (6) Mendeley (54)
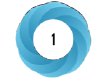	Lombardo EC, Lione R, Franchi L, Gaffuri F, Maspero C, Cozza P, Pavoni C. Dentoskeletal effects of clear aligner vs twin block-a short-term study of functional appliances. J Orofac Orthop. 2024 Sep;85(5):317-326. doi: 10.1007/s00056-022-00443-1.	Users-X (1) Mendeley (41)
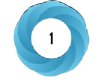	Knösel M, Espinoza-Espinoza GE, Sandoval-Vidal P, Zaror C. Angle class II correction: stepwise mandibular advancement or bite jumping? A systematic review and meta-analysis of skeletal, dental and condylar effects. J Orofac Orthop. 2020 Jul;81(4):286-300. English. doi: 10.1007/s00056-020-	Users-X (1) Mendeley (62)
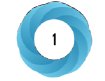	Chen Z, Chen Q, Fan X, Li Y, Mo S. Stepwise versus single-step mandibular advancement with functional appliance in treating class II patients : A meta-analysis. J Orofac Orthop. 2020 Sep;81(5):311-327. English. doi: 10.1007/s00056-020-00229-3.	Users-X (2) Mendeley (36)
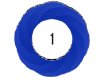	Medina AC. Functional appliance treatment for bilateral condylar fracture in a pediatric patient. Pediatr Dent. 2009 Sep-Oct;31(5):432-7.	Facebook Page (3) Mendeley (45)
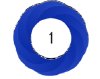	Von Bremen J, Erbe C, Pancherz H, Ruf S. Facial-profile attractiveness changes in adult patients treated with the Herbst appliance. J Orofac Orthop. 2014 May;75(3):167-74. English, German. doi: 10.1007/s00056-014-0210-3.	Facebook Page (1) Mendeley (32)
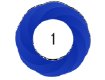	Pavoni C, Cretella Lombardo E, Lione R, Bollero P, Ottaviani F, Cozza P. Orthopaedic treatment effects of functional therapy on the sagittal pharyngeal dimensions in subjects with sleep-disordered breathing and Class II malocclusion. Acta Otorhinolaryngol Ital. 2017 Dec;37(6):479-485. doi: 10.14639/0392-100X-1420.	Facebook Page (2) Mendeley (32)
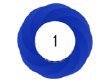	Li, Larry Ching Fan, and Ricky Wing-Kit Wong. “Management of severe Class II malocclusion with sequential removable functional and orthodontic appliances: A case for MOrthRCSEd* examination.” Dental Press Journal of Orthodontics 16 (2011): 1-11.	Facebook Page (1) Mendeley (12)

## DISCUSSION

Mandibular advancement devices are widely used in the treatment of Class II malocclusion with mandibular retrognathia and can increase mandibular length.^23^ The main results of this bibliometric analysis demonstrate that the topic is extensively researched, with over 30 years of research and a considerable number of citations, with emphasis on the Herbst appliance.

The oldest article on mandibular advancement devices is the Herbst appliance.^9^ This appliance was developed by Emil Herbst, who designed a fixed appliance to move the mandible forward after observing poor patient compliance with a removable appliance.^24^ However, after Pancherz’s publication,^11^ other mandibular advancement devices were developed, which may explain why the most cited article was also authored by Pancherz.^21^

The most cited article was published over 30 years ago, receiving a considerable number of citations (n = 352), which can be explained by the fact that it was a study that demonstrated the effectiveness of the Herbst appliance in the treatment of Class II malocclusion. Therefore, we can say that this is a classic article for the field, as it accumulates almost 400 citations, and for specific fields, articles with more than 100 citations are considered classics.^24^

Co-citation analysis revealed a highly structured and consolidated intellectual framework, dominated by classic studies primarily related to the Herbst appliance and the evaluation of mandibular growth. The prominence of the study of Pancherz et al.^11^ ^,^ ^21^ highlights their fundamental role in shaping the theoretical and methodological basis of research on mandibular advancement appliances. The dense interconnections between the groupings suggest conceptual continuity rather than fragmentation, indicating that contemporary studies continue to rely heavily on classic studies.

Bibliographic coupling analysis revealed distinct temporal patterns in the research field. Earlier foundational studies exhibited weaker coupling, reflecting heterogeneous reference bases characteristic of the area’s early development. In contrast, studies published from the late 1990s to the early 2000s formed a more cohesive network, suggesting methodological consolidation and shared research agendas.^4^ More recent publications appeared more peripherally connected and demonstrated weaker coupling, indicating that emerging topics, such as novel mandibular advancement devices,^12^ are being explored in a more fragmented manner and have not yet been fully integrated into the central research framework.

The most prevalent study design was clinical trials, followed by observational studies. This is justified by the fact that most studies aimed to evaluate skeletal, dental, and facial changes or compare two or more therapeutic approaches, suggesting that these two study designs would be more appropriate for this evaluation. The fact that most studies are clinical trials can be considered positive, since this methodology generally presents a higher level of evidence when compared to other designs, with randomized clinical trials being considered the gold standard in clinical evaluation, second only to systematic reviews of clinical trials in the evidence pyramid.^25^ However, the methodological quality of these studies must be carefully analyzed, as factors such as sample size, adequacy of randomization, blinding, bias control, and robustness of statistical analysis can directly influence the strength of the evidence produced.^26^

The most used mandibular advancement devices was the Herbst type. This may be partly explained by the fact that the Herbst appliance was one of the first mandibular propulsion devices described in the literature.^11^ Furthermore, Herbst and Twin-Block appear to be the mandibular advancement devices that have shown the greatest efficiency in mandibular growth, a fact that explains why they are the most frequently discussed.^4^

Analyzing the main objective of the articles, facial changes and aesthetic aspects were less frequent than skeletal and dental changes, despite being a predictor of treatment success.^27^ Some authors justify the lower incidence of this type of investigation due to the difficulty in obtaining this data.^28^ However, it seems that patients treated with mandibular advancement devices tend to have an improvement in facial attractiveness, contributing to this smaller overjets, more acute mental angles and no visible teeth at rest.^29^ ^,^ ^30^

Germany stood out for the number of publications, a fact associated with the origin of the main researcher in the field and their home university. Furthermore, the European continent demonstrates a tradition in scientific research, stemming from greater availability of financial resources and institutions historically focused on research, which explains the large volume of research, also reported by other authors.^29^ In addition, it is noticeable, as previously reported, that there tends to be a prominence in the number of publications in more economically favored countries, which provides a greater distribution of financial resources directed towards research.^31^

Although articles on mandibular advancement devices have a considerable number of citations in the academic environment, when we evaluate the social impact through altmetric analysis, we noticed a relatively low AAS and few articles with altmetric data available. This fact can be justified in part by the date of publication, where articles published a long time ago were included in the bibliometric analysis,^8^ ^,^ ^9^ and by the complexity of the study subject, which is not so easily understood by laypeople, who are the focus of social media.^32^ The social network that received the most mentions for this topic was Facebook, and this has already been reported in another altmetric analysis of orthodontic articles, and therefore, it seems that orthodontists tend to publish more results of their research on this social network than other dental specialties.^32^

This review’s strengths include its global scope, the absence of language or date restrictions, and the novelty of its bibliometric analysis of the topic. Its main limitation was the exclusive use of Web of Science, justified by the rigor in journal selection and detailed citation information, also adopted in other bibliometric reviews.^33^ ^-^ ^35^ Other databases, such as MEDLINE, Scopus, and Google Scholar, have limitations regarding citations, creation time, inclusion rigor, or the presence of unreviewed documents.^32^ Furthermore, citation analyses suffer from the lower number of recent articles and the lack of distinction between positive and negative citations. Another limitation of this study is that the review protocol was not prospectively registered in a public database. The absence of prior registration may increase the risk of methodological biases during the review process.

Based on bibliometric analyses, several topics appeared to be underrepresented in the literature. Specifically, there was little emphasis on studies evaluating changes in soft tissues and facial attractiveness, investigations involving newer mandibular advancement devices such as Invisalign Mandibular Advancement, long-term assessments of mandibular growth outcomes, and robust study designs, including clinical trials examining the interaction between mandibular advancement devices and growth hormone therapy. These findings suggest potential gaps and opportunities for future research.

## CONCLUSION

The analysis demonstrates that research on mandibular advancement devices has been extensively explored in the scientific literature, with a substantial body of studies evaluating these appliances, particularly in European countries, especially Germany and at the University of Giessen. The most frequently studied mandibular advancement appliance is the Herbst appliance, and most investigations focus on skeletal, dental, bone, and aesthetic outcomes. 

## Data Availability

All data generated or analyzed during this study are included in this published article.

## Supplementary Files

Supplementary file 1. Search strategy applied to the Web of Science in December of 2025.

[(((TS=(Power Scope device OR Twin Block OR Balters Bionator OR Jasper Jumper OR mandibular advancement Invisalign OR Herbst appliance OR Forsus Fatigue Resistance Device OR Cantilever Bite Jumper OR kinetor OR Kinetors OR Bimler Appliance OR Regulator, Frankel Function OR Function Regulator, Frankel OR Appliance, Herbst OR Herbst)) OR ALL=(Fixed functional appliance OR Flexible fixed functional appliances OR Rigid fixed functional appliances OR retruded mandible OR functional appliances OR Class II correctors OR retrognathic mandible OR fixed functional OR Orthodontics appliance, functional OR Functional Orthodontics Appliances OR Appliances, Functional Orthodontics OR Appliance, Functional Orthodontics))].

[Table t1app]

**Supplementary file 2. t1app:** Journals that most published about mandibular advancement devices.

Source Title	Number of articles	Impact factor
American Journal of Orthodontics and Dentofacial Orthopedics	174	3.0
European Journal of Orthodontics	93	2.7
Orthodontics and Craniofacial Research	19	1.7
International Orthodontics	13	1.9
Angle Orthodontist	11	3.2
Turkish Journal of Orthodontics	9	1.4
APOS Trends in Orthodontics	8	0.7
Korean Journal of Orthodontics	5	2.3
Progress in Orthodontics	5	5.0
Seminars in Orthodontics	6	2.0
